# Thermodynamics of Formation and Liquid–Vapor Phase Transitions of Antimony Alloys with Selenium and Sulfur

**DOI:** 10.3390/ma17010125

**Published:** 2023-12-26

**Authors:** Valeriy Volodin, Alina Nitsenko, Sergey Trebukhov, Xeniya Linnik, Yerkebulan Gapurov

**Affiliations:** Institute of Metallurgy and Ore Beneficiation JSC, Satbayev University, Almaty 050010, Kazakhstan; volodinv_n@mail.ru (V.V.); s.trebukhov@satbayev.university (S.T.); xenija_linnik@mail.ru (X.L.); erkebulan.gapurov@gmail.com (Y.G.)

**Keywords:** entropy, enthalpy, vapor pressure, melt, mixing, evaporation, boundaries, vapor–liquid equilibrium

## Abstract

The authors conducted liquid solution studies of antimony with selenium and sulfur in order to provide information on the thermodynamic functions of the formation of these alloys. The studies are based on the vapor pressure values of the components, comprising the double partial systems of antimony with antimony chalcogenides (Sb_2_Se_3_ and Sb_2_S_3_) and antimony chalcogenides with chalcogens (Se and S). We calculated the thermodynamic functions of mixing (graphical dependencies) and evaporation (tabular data) based on the partial vapor pressure values of components, which are represented by temperature–concentration dependencies. Based on the partial pressure values of melt components, we calculated the boundaries of liquid and vapor coexistence fields at atmospheric pressure (101.3 kPa) and in a vacuum (0.9 kPa). We established the absence of the stratification region on the Sb_2_S_3_–S diagram due to the fact that, on state diagrams, the stratification region is indicated at temperatures above 530 °C, while the boiling point of liquid sulfur at an atmospheric pressure corresponds to 429 °C. Based on the position of the field boundaries (L + V) on the state diagrams, the separation of antimony alloys with selenium and sulfur via distillation into elements at atmospheric pressure is difficult due to the high boiling points of antimony-based alloys in a vacuum: Sb_2_Se_3_–Se melts require some number of condensate re-evaporation cycles.

## 1. Introduction

Polymetallic matte (chalcogenide alloys) are obtained at copper, lead, and antimony plants in the processing polymetallic concentrates. Copper (Cu_2_S) and iron (FeS) sulfides form the basis of polymetallic mattes produced from copper and lead plants. Besides the main components, matte contains rare elements, particularly antimony in the form of compounds with sulfur, selenium, and tellurium, which isomorphically replace sulfur in sulfides. In this regard, various methods were developed and are being developed to remove compounds from the matte melt [1,2,3,4,5].

One of the methods intended to process mattes in order to improve their quality via the removal of lead and zinc sulfides is the distillation of volatile compounds in a vacuum at high temperatures. The distribution of rare sulfide elements among products during the distillation processing of mattes in a vacuum based on a very large number of studies and technological tests is provided and analyzed in [6]. A significant number of publications are devoted to the study of the thermodynamics of antimony melts with selenium and sulfur in relation to matte-processing conditions.

Several researchers were involved in the physical and chemical studies of liquid antimony–selenium alloys. The authors of [7] measured the kinematic viscosity and density of melts in the composition range from 40 mol.% Sb_2_Se_3_ + 60 mol.% Se to 20 mol.% Sb_2_Se_3_ + 80 mol.% Sb and from melting temperatures up to 1100–1200 °C.

In [8], the crystallization kinetics of glassy alloy Se_100−x_Sb_x_ (2 ≤ x ≤ 10) was studied via differential scanning calorimetry at different heating rates. The activation energy of the crystallization process, order parameter, rate constant, and frequency factor were determined. It was observed that chalcogenide glasses with a higher crystallization rate have lower thermal stability.

A significant number of studies using various methods [9,10,11,12,13,14,15,16,17,18] are devoted to the study of the composition and determination of the vapor pressure of components over the melts of the Sb–Se system, the determination of heat capacities and mixing functions, and the modeling of Sb_2_Se_3_ thermodynamics.

Thermodynamic studies of the antimony–sulfur system are also devoted to a considerable number of works [9,10,12,14,19,20], and they are summarized in monographs [21,22,23]. The authors of [11,24] in the studies of vapor compositions, with respect to antimony trisulfide, established the presence of SbS, S_2_, Sb_2_S_2_, Sb_2_S_2_, Sb_2_S_3_, Sb_2_S_4_, Sb_3_S_2_, Sb_3_S_3_, Sb_3_S_4_, Sb_3_S_4_, and Sb_4_S_5_ molecules, as well as fragment ions of different compositions. The thermodynamics of the quasi-double molten systems of antimony trisulfide with non-ferrous metal sulfides in mattes have been studied in [20,25,26,27,28,29].

In [20], the thermodynamic functions of the alloys of the Sb_2_S_3_–FeS system were determined using a calorimeter. The authors [25] studied the behavior of Sb_2_S_3_ in solid alloys with Cu_2_S [26], where the vapor pressure of antimony chalcogenide was determined at temperatures corresponding to the processes of matte vacuumization. In [27], on the basis of the saturated vapor values of antimony sulfide in the above system, a complete state diagram, including the boundaries of the liquid–vapor phase transition at atmospheric pressure and in a vacuum, was constructed. Based on it, the possibility of the separation of antimony chalcogenide into a vapor phase with its withdrawal into a condensate was stated.

The authors’ research in [28] is devoted to the study of the PbS–Sb_2_S_3_ system, where thermodynamic constants were obtained. Utilizing this research, the authors of [6] calculated the liquid–vapor phase transition in the lead sulfide–chalcogenide system on the basis of which the impossibility of the separation of the components of the system via distillation at atmospheric pressure was established. In [29], the researchers calculated the activities of the PbS–Sb_2_S_3_ components in a binary system via a modified model of the molecular interaction volume. The calculated results of the model agree with the experimental values, which allows, according to the authors, the provision of theoretical support for the vacuum separation and purification of PbS and Sb_2_S_3_.

In a more recent study [30], the authors obtained a set of self-consistent thermodynamic parameters of the S–Sb binary system and the Cu–S–Sb ternary system using the so-called CALPAD method of the associated model of liquid solutions. Based on the obtained data, they calculated the delamination regions of liquid solutions in the Sb–Sb_2_S_3_ and Sb_2_S_3_–S partial systems.

Analyzing the performed studies, it should be emphasized that despite the presence of a sufficiently large number of works devoted to the study of the melts of antimony with selenium and sulfur, the data on the thermodynamics of formation and evaporation are insufficient. The results of experimental determinations and calculations are inconsistent. Thus, in [20,31,32], the thermodynamic activity of selenium was found and calculated for a temperature of 994 K (721 °C), which is higher than the boiling point of solutions of this composition. In [30], the top of the dome of the two-phase liquid system Sb_2_S_3_–S corresponds to a temperature of ~1417 K (1144 °C) (determined by us graphically). The latter is erroneous, since the boiling point of sulfur does not exceed 703 K (430 °C), and the field of liquid solutions on the state diagram below the boiling point of sulfur is practically degenerate. In reference [33], in the private Sb_2_Se_3_–Se system, a significant part of the liquidus line is also above the boiling point line of selenium and antimony selenide solutions at atmospheric pressure.

There is no information on the vapor–liquid equilibrium for both systems. Currently, the most accurate method for determining the possibility of the separation of a binary system, in our case, Sb–Se and Sb–S, is to construct the boundaries of the vapor–liquid equilibrium based on the saturated vapor pressure values of the components of the system. The obtained data allow for judgments of the separation possibility of components (the number of “evaporation–condensation” cycles and the composition of the vapor phase) or its absence. Therefore, in this study, we present the results of determining the boundaries of melt and vapor coexistence in antimony–selenium and antimony–sulfur systems at atmospheric and low pressure, during which industrial matte distillation is usually conducted (Section 3.1 and Section 3.2). We also present the results of clarifying and determining the thermodynamic functions that are missing in the database of thermodynamic constants (Section 3.3).

## 2. Materials and Methods

### 2.1. Materials

The objects of study were the alloys of antimony with selenium and sulfur, and with the compositions specified in Table 1 and Table 2.

We prepared the alloys via the slow heating of antimony (99.99 wt.%) and sulfur (99.99 wt.%) (for Sb–S alloys) and antimony (99.99 wt.%) and selenium (99.99 wt.%), which was for Sb–Se alloys, in sealed evacuated quartz ampoules. The number of initial components corresponded to the composition of the specified alloy. Heating was performed at a rate of 50–100 °C per hour to a temperature 100 °C above the delamination area. The alloy was kept at this temperature for 12 h and quenched in water. The compositions of alloys numbered 5 corresponded to the stoichiometry of Sb_2_Se_3_ and Sb_2_S_3_ compounds.

### 2.2. Determining Thermodynamic Functions and the Boundaries of the Liquid–Vapor Phase Transition

The determination of thermodynamic functions in liquid Sb–Se and Sb–S systems is based on the partial pressure values of the components comprising the melts, which are presented in the form of temperature–concentration dependencies. The partial free mixing energy of Gibbs of the *i*–component of the system ($\Delta {\bar{G}}_{i}^{M}$) can be defined as $\Delta {\bar{G}}_{i}^{M}=-RT\ln a_{i}$, where *R* denotes the universal gas constant; *T* denotes temperature, K; and *a_i_* denotes the thermodynamic activity of the component in the solution. Activity (*a_i_*), in this case, is equal to the ratio of the partial pressure of the saturated vapor of the component above the solution (${\bar{p}}_{i}$) to the total vapor pressure above the liquid alloy.

The partial change in the component mixing entropy ($\Delta {\bar{S}}_{i}^{M}$) is determined via the differentiation of the dependence connecting the partial Gibbs free mixing energy with the following activity: ${\left(𝜕\Delta {\bar{G}}_{i}^{M}/𝜕T\right)}_{P}=-\Delta {\bar{S}}_{i}^{M}$. Then, the mixing enthalpy ($\Delta {\bar{H}}_{i}^{M}$) is determined via the $\Delta {\bar{H}}_{i}^{M}=\Delta {\bar{G}}_{i}^{M}+T\Delta {\bar{S}}_{i}^{M}$ equation. The integral mixing functions of alloys are defined as the sum of the parts of partial mixing functions.

The partial entropies of the evaporation of melt components ($\Delta {\bar{S}}_{i}^{V}$) are found via the differentiation of the partial Gibbs evaporation energy that is equal to $\Delta {\bar{G}}_{i}^{V}=-RT\ln{\bar{p}}_{i}$. The partial enthalpies of vaporization are obtained as $\Delta {\bar{H}}_{i}^{V}=\Delta {\bar{G}}_{i}^{V}+\Delta {\bar{S}}_{i}^{V}$. The integral thermodynamic functions of evaporation are calculated as the sum of the parts of partial values.

The determination of the boundaries of the liquid–vapor phase transition in systems containing chalcogens and chalcogenides (the boiling point and the corresponding composition of the vapor phase) is complicated by the following: high boiling temperatures of solutions; the difficulty of determining the concentration of components in the vapor phase that are in equilibrium with the alloy; problems with ebulliometric measurement equipment due to the aggressiveness of vapors relative to equipment materials.

Liquid chalcogenide solutions do not boil due to the high density of their constituent component. Therefore, the boiling point was determined to be equal to the temperature at which the sum of the partial vapor pressures of the system’s components under Dalton’s law is equal to atmospheric (101.3 kPa) or other pressures corresponding to the conditions of vacuum technologies. Thus, the temperature–concentration dependencies of the partial pressure of elements and compounds are required to calculate phase boundaries.

The composition of the vapor phase (concentration of the *i*-component—$y_{i}$—and *j*-component—$y_{j}$) above the melts was determined using the well-known Equation (1):

(1) $$y_{i}(y_{j})[mole\;fraction]=n_{i}(n_{j})/(n_{i}+n_{j})={\bar{p}}_{i}({\bar{p}}_{j})/({\bar{p}}_{i}+{\bar{p}}_{j}),$$

where $n_{i}$ and $n_{j}$ are the number of moles of the *i*-component and *j*-component in the vapor phase; and ${\bar{p}}_{i}$ and ${\bar{p}}_{j}$ are the partial vapor pressures of the *i*-component and *j*-component in the vapor phase, Pa.

### 2.3. Determining the Partial Pressure of Vapor Components

Since there are compounds Sb_2_Se_3_ and Sb_2_S_3_ in Sb–Se and Sb–S melts [30,33] that melt congruently, with each having its own melting and boiling points, these systems were studied as two particular systems: Sb–Sb_2_Se_3_ and Sb_2_Se_3_–Se for the first system and Sb–Sb_2_S_3_ and Sb_2_S_3_–S for the second system.

It was previously established that the saturated vapor pressure values of the more volatile components are as follows: Sb_2_Se_3_ and Sb_2_S_3_ (in Sb–Sb_2_Se_3_ and Sb–Sb_2_S_3_ systems, respectively) and Se and S (in Sb_2_Se_3_–Se and Sb_2_S_3_–S systems, respectively) are more than two orders higher than the pressure of less volatile components. In this regard, we chose the boiling-point method (isothermal version) as a method for determining the vapor pressure value. The method is based on a significant increase in the evaporation rate when the external and saturated vapor pressures of the substance are equal to a decrease in pressure above the melt and a particular temperature. This method does not require knowledge of the molecular mass of the vapor, which introduces errors in the calculations of other methods. In this case, we considered that the vapor phase consists entirely of the more volatile *i*-component.

**Figure 1:**
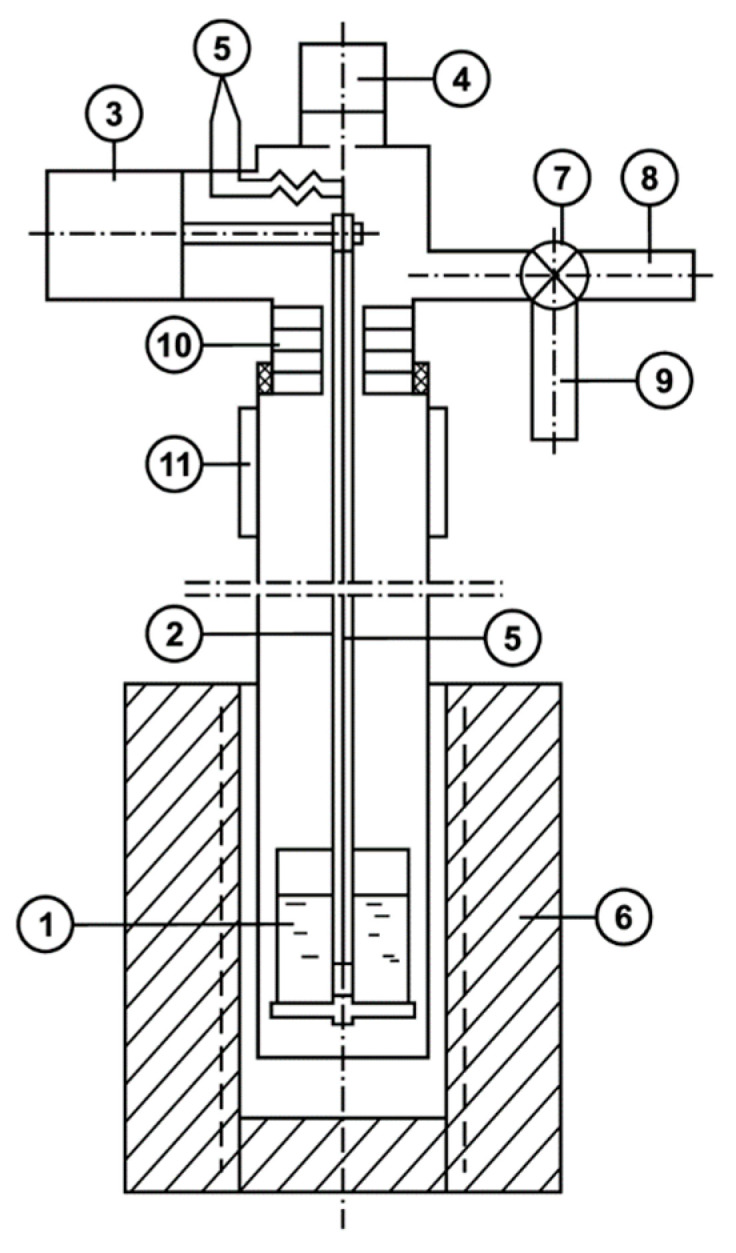
Scheme of the installation for determining vapor pressure: (1) Quartz crucible; (2) Quartz suspension; (3) Weight measurement system; (4) Pressure measurement system; (5) Thermocouple; (6) Electric furnace; (7) Leak valve; (8) Gas evacuation line; (9) Inert gas supply line; (10) Shield; (11) Caisson.

The unit scheme intended to determine vapor pressure using the boiling-point method is shown in Figure 1. The unit is a retort made of two parts: the lower part, placed in an electric furnace with automatic temperature maintenance; and the upper part, made of quartz glass. A crucible with a portion of the alloy is mounted on a hollow suspension inside the retort. There is a junction of a platinum–platinum–rhodium thermocouple inside the suspension at the melt level in the crucible.

The suspension rests on the scales of the mass loss measurement system located in the upper part of the retort. The parts of the retort are articulated using a rubber seal that is removed from the high-temperature zone. The lower and upper parts of the retort are separated by screens to reduce heat flow from the high-temperature zone. There is a pressure measurement system, lines for gas evacuation, argon filling, and thermocouple end outlets in the upper part of the retort. Systems for the measurement of mass loss, pressure, and temperature have signals that are outputted to a multipoint potentiometer, which records measurements on a chart tape.

The experimental procedure was as follows. We placed an alloy sample (up to 2 g) in a crucible mounted on a suspension with the retort disconnected outside the heating zone. Then, we articulated the lower part of the retort with the upper one. The retort was evacuated twice with a vacuum pump 2HVR-5DM UHL4 (Vacuummash, Kazan, Russia) and filled with argon. Then, we placed the lower part of the retort in the isothermal zone of a preheated electric furnace RT 50/250/13 (Nabertherm, Bremen, Germany). We heated the retort at an excess pressure of 2–5 kPa with an open inert gas supply system to suppress the evaporation process of components and compensate for the increase in pressure in the retort due to gas expansion during heating. When the alloy sample reached the specified temperature, we began the evacuation of argon from the retort with a constant alloy temperature (isothermal option). At the same time, we recorded the mass loss of the sample and the change in pressure simultaneously. We considered that the pressure at which a sharp increase in the evaporation rate was observed was equal to the saturated vapor pressure of the *i*-component above the alloy.

The processing of the results and the determination of the saturated vapor pressure value are described in detail in our work [34].

The dependence of the vapor pressure value in the $\ln{\bar{p}}_{i}-T^{-1}$ coordinate under the Arrhenius equation corresponds to a linear dependence. Thus, we performed the experimental determination for the boundary temperatures of the interval where it is possible to obtain boiling-point values that are significant for the method. In this case, we repeated the experiments three times under similar conditions for each temperature and alloy composition.

We calculated the dependence of the vapor pressure on temperature in the form of the $\ln{\bar{p}}_{i}=B-A\times T^{-1}$ equation for each alloy composition. Then, for each system, we obtained the temperature–concentration dependence of the saturated vapor pressure of the *i*-component ($\ln{\bar{p}}_{i}=B_{\left(x_{i}\right)}-A_{\left(x_{i}\right)}\times T^{-1}$, where *x_i_* is the mole fraction of the *i*-component in the alloy, unit fractions) based on the polynomials of the dependence of Coefficients A and B from the alloy composition.

We determined the value of the partial pressure of the saturated vapor of the slightly volatile *j*-component above the alloy as ${\bar{p}}_{j}=p_{j}^{o}\times a_{j}=p_{j}^{o}\times \gamma_{j}\times x_{j}$, where $p_{j}^{o}$ denotes the saturated vapor pressure over the pure *j*-component; $a_{j}$ denotes the activity of the *j*-component; $\gamma_{j}$ denotes the activity coefficient of the *j*-component; and $x_{j}$ denotes the concentration of the *j*-component in the alloy, which is equal to $x_{j}=1-x_{i}$.

The activity coefficient of the low-volatile component ($\gamma_{j}$) was calculated via the numerical integration of the Gibbs–Duhem equation using the auxiliary function $\alpha_{i_{3}}=\ln\gamma_{i}/x_{j}^{2}$ proposed by Darken [35]. After transformation [36] and substitution into the equation $\ln\gamma_{j}=-\int_{\ln\gamma_{i}\;at\;x_{j}=1}^{\ln\gamma_{i}\;at\;x_{j}}\frac{x_{i}}{x_{j}}d\ln\gamma_{i}$, it connects $\ln\gamma_{i}$ and $\ln\gamma_{j}$ in the form of an expression that is convenient for numerical integration:

(2) $$\ln\gamma_{j}=-\frac{\ln\gamma_{i}\times x_{i}\times x_{j}}{x_{j}^{2}}+\int_{x_{i}=0}^{x_{i}}\frac{\ln\gamma_{i}}{{\left(1-x_{i}\right)}^{2}}dx_{i}.$$

In Equation (2), the activity coefficient of the *i*-component in the alloy ($\gamma_{i}$) is obtained from the following expression:

(3) $$\ln\gamma_{i}=\ln{\bar{p}}_{i}-\ln p_{i}^{o}-\ln x_{i},$$

where $p_{i}^{o}$ denotes the saturated vapor pressure above the pure *i*-component, Pa; and $x_{i}$ denotes the mole fraction of the *i*-component in the alloy in unit fractions.

## 3. Results and Discussion

### 3.1. Determination of the Saturated Vapor Pressure of Melt Components

#### 3.1.1. Sb–Se System

Considering a large amount of a priori information about the Sb–Se system, we believed that the vapor above the melts of the particular Sb–Sb_2_Se_3_ system consists of antimony selenide, and it is represented by selenium above the Sb_2_Se_3_–Se melts. The results of the experimental partial pressure determinations of the saturated vapor of antimony selenide (${\bar{p}}_{Sb_{2}Se_{3}}$ experiment) in the Sb–Sb_2_Se_3_ system and selenium (${\bar{p}}_{Se}$ experiment) in the Sb_2_Se_3_–Se system, as well as the calculated values of the vapor pressure of antimony (${\bar{p}}_{Sb}$ calculation) in the first system and antimony selenide (${\bar{p}}_{Sb_{2}Se_{3}}$ calculation) in the second system, are specified in Table 3 and Table 4. Alloy compositions, temperatures (T and K) for the experiments, and the relative error of approximation (Δ, rel.%) for experimentally obtained data are also provided. The total measurement error is defined as the sum of the errors of independent measurements: temperature—1%; weight—0.1%; pressure—0.5%; experimental data approximation—3.88%; and equal to 5.48%.

The values of the partial pressure of the saturated vapor of antimony selenide over alloys with antimony (${\bar{p}}_{Sb_{2}Se_{3}}$), determined experimentally, are provided in Table 3 and approximated via the following dependence:

(4) $$\begin{array}{c} \ln{\bar{p}}_{Sb_{2}Se_{3}}[Pa]=(-6358x_{Sb_{2}Se_{3}}^{4}+16,234x_{Sb_{2}Se_{3}}^{3}-10,839x_{Sb_{2}Se_{3}}^{2}-1990_{Sb_{2}Se_{3}}-14,057)\cdot T^{-1}+\\ +4.961x_{Sb_{2}Se_{3}}^{4}-11.959x_{Sb_{2}Se_{3}}^{3}+8.019x_{Sb_{2}Se_{3}}^{2}+0.379x_{Sb_{2}Se_{3}}+22.864+\ln x_{Sb_{2}Se_{3}} \end{array},$$

where $x_{Sb_{2}Se_{3}}$ denotes the mole fraction of antimony selenide in the melt as equal to the following: $0\leq x_{Sb_{2}Se_{3}}\leq 1$.

We determined the saturated vapor pressure of liquid antimony selenide as Equation (5), and the estimated values of saturated antimony vapor in the Sb–Sb_2_Se_3_ system correspond to Equation (6), where $0\leq x_{Sb}\leq 1$ denotes the mole fraction of antimony in the alloy.

(5) $$\ln p_{Sb_{2}Se_{3}}^{o}[Pa]=-17,010\cdot T^{-1}+24.264.$$

(6) $$\begin{array}{c} \ln{\bar{p}}_{Sb}[Pa]=(-6358x_{Sb}^{4}+17,675x_{Sb}^{3}-14,082x_{Sb}^{2}+968x_{Sb}-14,958-398\ln x_{Sb})\cdot T^{-1}+\\ +4.961x_{Sb}^{4}-14.5x_{Sb}^{3}+13.735x_{Sb}^{2}-4.2x_{Sb}+20.312+1.384\ln x_{Sb} \end{array}.$$

The total measurement error for the Sb_2_Se_3_–Se (Table 4) system is determined as the sum of the errors of independent measurements, and it is equal to 4.72%. In accordance with the obtained experimental data, we approximated the partial pressure values for saturated selenium vapor (${\bar{p}}_{Se}$) over alloys with antimony selenide via the dependence (Equation (7)), where $x_{Se}$ denotes the mole fraction of selenium in the melt as equal to the following: $0\leq x_{Se}\leq 1$. We also approximated that the partial pressure of antimony selenide for system Sb_2_Se_3_–Se corresponds to Equation (8).

(7) $$\begin{array}{c} \ln{\bar{p}}_{Se}[Pa]=(1042x_{Se}^{3}-2226x_{Se}^{2}+446x_{Se}-11,771)\cdot T^{-1}-\\ -0.583x_{Se}^{3}+2.132x_{Se}^{2}-1.589x_{Se}+24.803+\ln x_{Se} \end{array}.$$

(8) $$\begin{array}{c} \ln{\bar{p}}_{Sb_{2}Se_{3}}[Pa]=(-1042x_{Sb_{2}Se_{3}}^{3}+2463x_{Sb_{2}Se_{3}}^{2}-920_{Sb_{2}Se_{3}}-17,511-880\ln x_{Sb_{2}Se_{3}})\cdot T^{-1}+\\ +0.583x_{Sb_{2}Se_{3}}^{3}-0.491x_{Sb_{2}Se_{3}}^{2}-1.692x_{Sb_{2}Se_{3}}+25.864+1.926\ln x_{Sb_{2}Se_{3}} \end{array}.$$

#### 3.1.2. Sb–S System

When experiments are conducted with melts of the Sb–S system, we accepted that the vapor above the melts of the particular Sb–Sb_2_S_3_ system consists of antimony sulfide. Above Sb_2_S_3_–S melts, the vapor is represented by sulfur. The results of the experimental partial pressure determinations of the saturated vapor of antimony sulfide (${\bar{p}}_{Sb_{2}S_{3}}$ experiment) and sulfur (${\bar{p}}_{S}$ experiment) in the Sb_2_S_3_–S system, as well as the calculated values of antimony vapor pressure (${\bar{p}}_{Sb}$ calculation) in the first system, are provided in Table 5 and Table 6. Alloy compositions, temperatures (T and K) for the experiments, and the relative error (Δ, rel.%) of approximation for experimentally obtained data are also provided in Table 5 and Table 6. The total measurement error is determined as the sum of the errors of independent measurements for the Sb–Sb_2_S_3_ system, which is equal to 4.94%.

For this system, we approximated the partial pressure values for the saturated vapor of antimony sulfide (${\bar{p}}_{Sb_{2}S_{3}}$) over alloys with antimony via the following dependence:

(9) $$\begin{array}{c} \ln{\bar{p}}_{Sb_{2}S_{3}}[Pa]=(-1034x_{Sb_{2}S_{3}}^{3}+5255x_{Sb_{2}S_{3}}^{2}-9635x_{Sb_{2}S_{3}}-13459)\cdot T^{-1}+\\ +0.097x_{Sb_{2}S_{3}}^{3}-0.967x_{Sb_{2}S_{3}}^{2}+3.313x_{Sb_{2}S_{3}}+22.574+\ln x_{Sb_{2}S_{3}} \end{array},$$

where $x_{Sb_{2}S_{3}}$ is a mole fraction of antimony sulfide in the melt, and it is equal to the following: $0\leq x_{Sb_{2}S_{3}}\leq 1$.

The saturated vapor pressure of liquid antimony sulfide is determined by us as Equation (10), and the calculated values of saturated antimony vapor in the Sb–Sb_2_S_3_ system—Equation (11), where $0\leq x_{Sb}\leq 1$ is a mole fraction of antimony.

(10) $$\ln p_{Sb_{2}S_{3}}^{o}[Pa]=-18,873\cdot T^{-1}+25.017.$$

(11) $$\begin{array}{c} \ln{\bar{p}}_{Sb}[Pa]=(1034x_{Sb}^{3}+602x_{Sb}^{2}-2079x_{Sb}-16312-2227\ln x_{Sb})\cdot T^{-1}-\\ -0.097x_{Sb}^{3}-0.531x_{Sb}^{2}-0.318x_{Sb}+21.254+2.67\ln x_{Sb} \end{array}.$$

Table 6 shows the coefficients of the Arrhenius equation for sulfur vapor pressure and the boiling point of solutions at atmospheric pressure (101.325 kPa); 0.9 kPa is the pressure at which, as a rule, the vacuum thermal processing of mattes is performed. As can be seen, the partial pressure values of the saturated vapor of sulfur remain almost constant throughout the entire concentration range of the pseudobinary Sb_2_S_3_–S system, and they are equal to the vapor pressure value above elemental sulfur, $\ln p_{S}^{o}[Pa]=-8811\cdot T^{-1}+24.081$, as evidenced by the constancy of boiling temperatures. Some deviations of the boiling temperature for the melt from that of sulfur are less than the experimental error. This is because the field of liquid solutions at the sulfur edge of the phase diagram is degenerate, and the liquidus line is located close to the sulfur ordinate. Antimony sulfide Sb_2_S_3_ crystallizes from the solution when the liquidus line is crossed, but the composition of the liquid phase remains constant; only the ratio of the crystalline and liquid phases changes. The boiling point of solutions is, in this case, practically an isotherm.

### 3.2. Liquid–Vapor Phase Transition in the Systems of Antimony with Selenium and Sulfur

**Figure 2:**
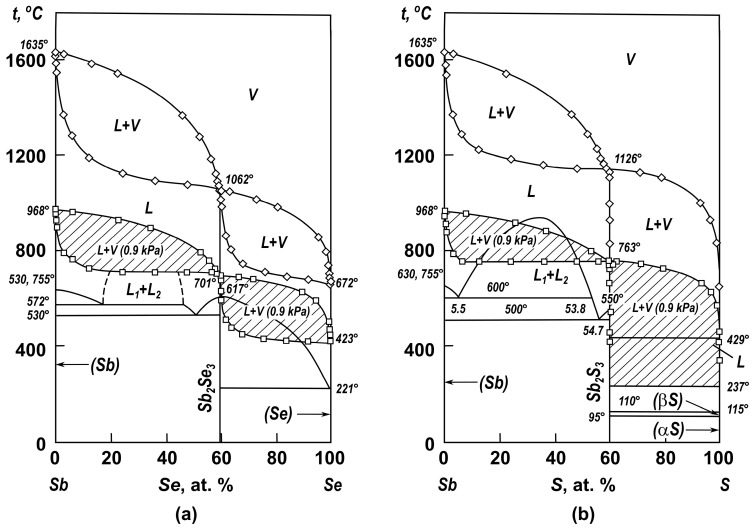
Complete state diagram of the antimony–selenium (**A**) and antimony–sulfur (**B**) systems.

We calculated the boundaries of the coexistence fields of liquid and vapor in the Sb–Se and Sb–S systems based on the partial pressure values for the vapor of components in the molten systems of antimony with selenium and sulfur and in accordance with the dependencies provided above (Equations (4)–(11)). The field boundaries (*L + V*) at atmospheric pressure (101.325 kPa) and in a vacuum (0.9 kPa) (shaded) are plotted on the phase diagrams (Figure 2).

Considering the position of the boundaries of the coexistence fields of liquid and vapor at atmospheric pressure and in a vacuum, we can observe that antimony cannot be completely purified from antimony selenide (Figure 2A) and sulfide (Figure 2B) via distillation in a vacuum in one operation. This consequence is due to the small size of the field (*L + V*) with respect to temperature. Several “evaporation–condensation” operations are required for a sufficiently complete purification of antimony.

When selenium and antimony selenide are separated in a vacuum (Figure 2A) from the region of melts adjacent to the antimony selenide, the melt and vapor coexistence field (*L + V*) is superimposed on the two-phase region (Sb_2_Se_3 crystal._ + *L*). That is, the liquidus line in this particular system for alloys that are rich in antimony selenide, as shown in the known phase diagrams [30,33], is not correct. However, it will not cause technological difficulties: the vapor phase is almost completely represented by selenium. Via the distillation of selenium from melts in vacuum (0.9 kPa) within the entire concentration range of the Sb_2_Se_3_–Se system, a mixture of solution with crystals (Sb_2_Se_3 crystal._) is produced, with the accumulation of the latter in the cube residue.

For the Sb–Sb_2_S_3_ system (Figure 2B), the coexistence field of melt and vapor at low pressures (*L + V* (0.9 kPa)) is superimposed on the two-phase region of the separation of liquid solutions (*L*_1_ + *L*_2_), as evidenced by the boiling point of liquid solutions, which is practically an isotherm. In the Sb_2_S_3_–S system, the two-phase coexistence field of liquid and crystalline antimony sulfide Sb_2_S_3 crystal._ is limited by the boiling point of the liquid (sulfur) at 429 °C and at atmospheric pressure, which excludes the existence of the delamination region provided by the authors of [30]. The separation of sulfur and antimony sulfide at atmospheric pressure and in a vacuum does not imply technological difficulties: the vapor phase is almost completely represented by sulfur. When selenium is distilled from melts in a vacuum (0.9 kPa), throughout the entire concentration range of the Sb_2_Se_3_–Se system, it will flow from a mixture of solution with crystals (Sb_2_Se_3 crystal._), with the latter accumulating in the distillation residue.

Based on the obtained data, we can draw the following conclusion. From a technological point of view, the purification of selenium and sulfur from antimony via the distillation of chalcogen in a vacuum will not cause difficulties. Selenium and antimony selenide and sulfur and antimony sulfide are completely converted into vapor and then into sulfide condensates under the vacuum distillation processing conditions of mattes at temperatures above 1100–1200 °C.

### 3.3. Thermodynamics of the Formation of Antimony with Selenium and Sulfur Solutions

**Figure 3:**
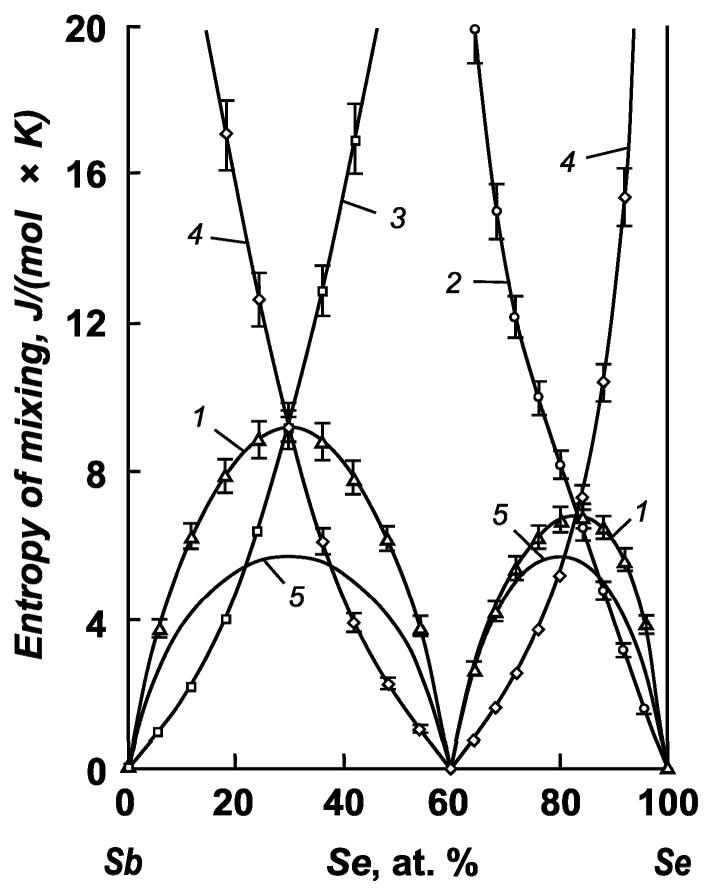
Integral (1, 5) and partial (2–4) mixing entropies of the components of antimony–selenium melts: 1—Sb–Se system; 2—selenium; 3—antimony; 4—antimony selenide; 5—ideal system.

**Figure 4:**
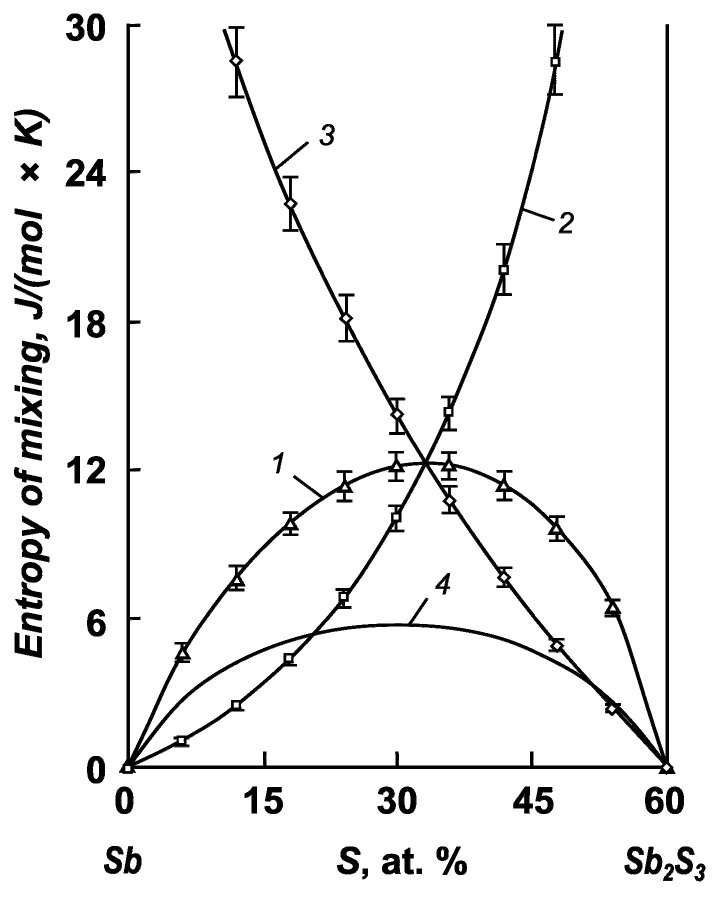
Integral (1, 4) and partial (2, 3) mixing entropies of the components of antimony–sulfur melts: 1—Sb–Sb_2_S_3_ system; 2—antimony; 3—antimony sulfide; 4—ideal system.

The calculated partial and integral mixing entropies of the components of the antimony–selenium and antimony–sulfur systems are shown in Figure 3 and Figure 4.

Analyzing the dependencies, we can observe that the integral mixing entropies of alloys in the antimony–antimony selenide system have a positive maximum. The formation of alloys is accompanied by an increase in disorder in the system. The extremum of the integral entropy of mixing reaches a value of 9.18 ± 0.50 J/(mol × K) at a concentration of 30 at. % Se in the melt. In the Sb_2_Se_3_–Se system, the maximum entropy of mixing corresponds to a value of 6.84 ± 0.34 J/(mol × K) at a content of 83.5–83.6 at. % selenium in the solution.

For the antimony–sulfur system, we defined mixing functions only for the particular Sb–Sb_2_S_3_ system (0–60 at. % S). This is because the field of liquid solutions in the Sb_2_S_3_–S system (60–100 at. % S) below the boiling point of sulfur is degenerate, and the liquid bath consists of almost pure sulfur and antimony sulfide crystals. Here, the formation of liquid alloys is also accompanied by disorder. The maximum of the integral mixing entropy corresponds to a composition of 34.3 at. % S and a value of 12.26 ± 0.61 J/(mol × K).

The integral entropy of mixing significantly exceeds that of an ideal system in both antimony systems with selenium and sulfur. This indicates a significant amount of excess entropy with respect to mixing and correlates with phase diagrams where regions of separation are present.

**Figure 5:**
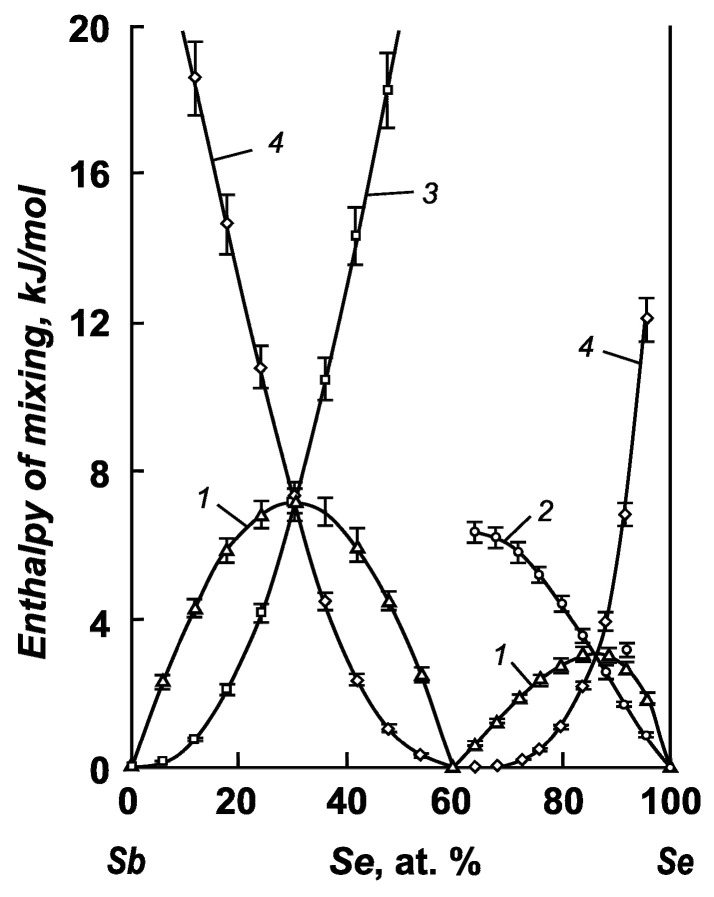
Integral (1) and partial (2–4) mixing enthalpies of the components of antimony–selenium melts: 1—Sb–Se system; 2—selenium; 3—antimony; 4—antimony selenide.

**Figure 6:**
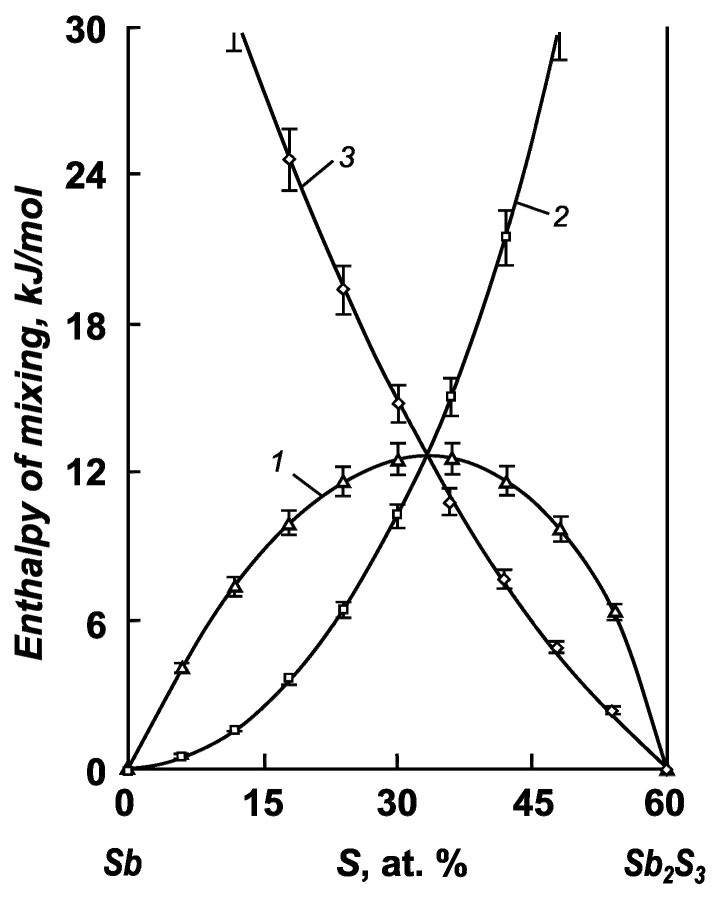
Integral (1) and partial (2, 3) enthalpies of the mixing components of antimony–sulfur melts: 1—Sb–Sb_2_S_3_ system; 2—antimony; 3—antimony sulfide.

The integral mixing enthalpy of antimony and selenium has a noticeable positive value (7.19 ± 0.40 kJ/mol) at 30 at. % selenium, and a value of 3.16 ± 0.15 kJ/mol is observed at 86.7 at. % selenium in the melt (Figure 5). In the antimony–antimony sulfide system, the maximum integral enthalpy of mixing reaches a value of 12.74 ± 0.63 kJ/mol at 31.5 at. % S (Figure 6). A positive value of the integral enthalpy of mixing indicates the absence of interactions between particles in the liquid bath.

### 3.4. Thermodynamics of the Evaporation of Antimony Solutions with Selenium and Sulfur

We observed the partial entropies of the evaporation of components in partial systems of antimony with selenium and sulfur via the differentiation of the partial Gibbs evaporation energy by temperature and the integral entropies by summing the shares of partial functions.

The values of the partial entropies of the evaporation of melt components [antimony ($\Delta {\bar{S}}_{Sb}^{V}$), selenium ($\Delta {\bar{S}}_{Se}^{V}$), and antimony selenide ($\Delta {\bar{S}}_{Sb_{2}Se_{3}}^{V}$)] of the antimony–selenium system and sulfur ($\Delta {\bar{S}}_{S}^{V}$) and antimony sulfide ($\Delta {\bar{S}}_{Sb_{2}S_{3}}^{V}$) of antimony–sulfur melts, as well as integral functions ($\Delta S_{Sb-Se}^{V}$) and ($\Delta S_{Sb-S}^{V}$), are summarized in Table 7 and Table 8.

As shown in Table 7 and Table 8, the change in the evaporation entropy of Sb_2_Se_3_ was 105.93 ± 5.12 J/(mol × K), and it was 112.17 ± 5.54 J/(mol × K) with respect to Sb_2_S_3_. The integral evaporation entropy value from antimony to compounds Sb_2_Se_3_ and Sb_2_S_3_ increases and remains almost constant for Sb_2_Se_3_–Se melts. The $\Delta S_{Sb-S}^{V}$ of the liquid alloys of antimony with sulfur is equal to the evaporation entropy of elemental sulfur.

The values of the partial enthalpies of the evaporation of melt components [antimony ($\Delta {\bar{H}}_{Sb}^{V}$), selenium ($\Delta {\bar{H}}_{Se}^{V}$), and antimony selenide ($\Delta {\bar{H}}_{Sb_{2}Se_{3}}^{V}$)] of the antimony–selenium system and sulfur ($\Delta {\bar{H}}_{S}^{V}$) and antimony sulfide ($\Delta {\bar{H}}_{Sb_{2}S_{3}}^{V}$) of antimony–sulfur alloys, as well as integral functions ($\Delta H_{Sb-Se}^{V}$) and ($\Delta H_{Sb-S}^{V}$), are summarized in Table 9 and Table 10. The change in the enthalpy of the evaporation of Sb_2_Se_3_ amounted to 141.42 ± 6.68 kJ/mol, and it was 156.92 ± 7.75 kJ/mol with respect to Sb_2_S_3_. The integral value of the enthalpy of vaporization increases from antimony to Sb_2_Se_3_ and Sb_2_S_3_ compounds; in the Sb_2_Se_3_–Se system, it decreases as the liquid composition approaches the selenium edge of the state diagram. The $\Delta H_{Sb-S}^{V}$ of the liquid alloys of antimony with sulfur is equal to the vaporization enthalpy of elemental sulfur—73.26 ± 3.62 kJ/mol.

The obtained thermodynamic data will supplement information on the thermodynamic values of the liquid alloys of antimony with selenium and sulfur.

## 4. Conclusions

We performed presented thermodynamic studies of liquid antimony solutions with selenium and sulfur due to the lack of information on the liquid alloys of antimony–selenium and antimony–sulfur systems. Two particular state diagrams were considered in the first system: Sb–Sb_2_Se_3_ and Sb_2_Se_3_–Se. Sb–Sb_2_S_3_ and Sb_2_S_3_–S were considered in the second system. The studies were based on the vapor pressure values of the components of particular systems. We determined the vapor pressure of highly volatile components via the boiling-point method (isothermal version), as well as the vapor pressure of less volatile components via the numerical integration of the Gibbs–Duhem equation. In this study, we represented the obtained partial vapor pressures of the components by temperature-concentration dependencies.

The thermodynamic functions of the formation and evaporation of liquid solutions in the Sb–Sb_2_Se_3_, Sb_2_Se_3_–Se, Sb–Sb_2_S_3_, and Sb_2_S_3_–S systems were calculated by us using known dependencies based on the partial values of the vapor pressure of the components in the systems. Thermodynamic mixing functions are presented graphically; evaporation functions are tabulated.

We defined that the integral mixing entropy of alloys in the Sb–Sb_2_Se_3_ system is accompanied by an increase in disorder within the system. The extremum of the function reaches a value of 9.18 ± 0.50 J/(mol × K) at a concentration of 30 at. % Se in the melt. In the Sb_2_Se_3_–Se particular system, the maximum entropy of mixing corresponds to a value of 6.84 ± 0.34 J/(mol × K) at contents of 83.5–83.6 at. % selenium in the solution. Due to the degeneracy of the field of liquid solutions in the Sb_2_S_3_–S system at the boiling point of sulfur, we determined the mixing functions only for the particular Sb–Sb_2_S_3_ system (0–60 at. % S). Here, the formation of liquid alloys is also accompanied by disorder. The maximum integral entropy of mixing corresponds to a composition of 34.3 at. % S and a value of 12.26 ± 0.61 J/(mol × K). We established that the integral entropy of mixing significantly exceeds that of an ideal system in both antimony systems with selenium and sulfur. This indicates a significant amount of excess mixing entropy.

In this study, we also defined that the integral mixing enthalpy of antimony and selenium has a noticeable positive value: 7.19 ± 0.40 kJ/mol at 30 at. % Se and 3.16 ± 0.15 kJ/mol at 86.7 at. % Se. The maximum integral mixing enthalpy in the Sb–Sb_2_S_3_ system reaches a value of 12.74 ± 0.63 kJ/mol at 31.5 at. % S. A positive integral mixing enthalpy value indicates the absence of interactions between particles in the liquid bath. The change in the evaporation enthalpy of the congruently melting Sb_2_Se_3_ compound amounted to 141.42 ± 6.68 kJ/mol, and the change in Sb_2_S_3_ amounted to 156.92 ± 7.75 kJ/mol. The integral value of the enthalpy of vaporization from antimony to Sb_2_Se_3_ and Sb_2_S_3_ compounds increases; in the Sb_2_Se_3_–Se system, the value decreases as the liquid composition approaches the selenium edge of the state diagram. The evaporation enthalpy of liquid Sb_2_Se_3_–S alloys is equal to the evaporation enthalpy of elemental sulfur: 73.26 ± 3.62 kJ/mol.

Based on the partial pressure values of melt components, we calculated the boundaries of liquid and vapor coexistence fields at atmospheric pressure (101.3 kPa) and in a vacuum of 0.9 kPa, at which the vacuum distillation process of industrial mattes was realized. We established that the liquidus line did not correspond to the known state diagrams in the Sb_2_Se_3_–Se system, and there was no delamination region in the Sb_2_Se_3_–S diagram. Based on the position of the field boundaries (*L + V*) on the supplemented state diagrams, the separation of antimony alloys with selenium and sulfur distillation into components at atmospheric pressure is difficult due to the high boiling temperatures of antimony-based alloys. It requires repeated condensate re-evaporation cycles in a vacuum.

The results of the study will be of interest to metallurgical technicians engaged in the processing of polymetallic matte and the extracting of rare elements from such raw materials, as well as specialists in the field of physical chemistry.

## Figures and Tables

**Table 1:** Composition of alloys in the Sb–Se system.

Alloy Number	Alloy Composition, Mass %		Alloy Composition, at. %	
	Se	Sb	Se	Sb
1	10.14	89.86	14.82	85.18
2	18.83	81.17	26.35	73.65
3	26.11	73.89	35.27	64.73
4	36.99	63.01	47.51	52.49
5	49.31	50.69	60.00	40.00
6	59.03	40.97	68.96	31.04
7	66.49	33.51	75.37	24.63
8	76.49	23.51	83.38	16.62
9	87.19	12.81	91.30	8.70

**Table 2:** Composition of alloys in the Sb–S system.

Alloy Number	Alloy Composition, Mass %		Alloy Composition, at. %	
	S	Sb	S	Sb
1	5.63	94.37	18.47	81.53
2	12.00	88.00	34.11	65.89
3	15.51	84.49	41.07	58.93
4	20.41	79.59	49.33	50.67
5	28.32	71.68	60.00	40.00
6	35.28	64.72	67.43	32.57
7	45.60	54.40	76.09	23.91
8	56.83	43.17	83.33	16.67
9	80.83	19.17	94.12	5.88

**Table 3:** Vapor pressure of antimony selenide and antimony in the Sb–Sb_2_Se_3_ system.

Alloy Composition, at. %		*T*, K	${\bar{p}}_{Sb_{2}Se_{3}}$, Experiment, kPa	${\bar{p}}_{Sb_{2}Se_{3}}$, Calculation, kPa	Δ, rel. %	${\bar{p}}_{Sb}$, Calculation, kPa
Se	Sb					
14.82	85.18	1073	2.53	2.75	−8.00	9 × 10^−2^
			2.93		+6.54	
			2.80		+1.82	
		1273	24.53	24.65	−0.49	1.00
			25.33		+2.76	
			23.13		−2.11	
26.35	73.65	1073	3.20	3.37	−5.04	8 × 10^−2^
			3.60		+6.82	
			3.33		−1.19	
		1273	34.26	34.51	−0.72	0.85
			34.66		+0.43	
			34.66		+0.43	
35.27	64.73	1073	3.33	3.55	−6.20	8 × 10^−2^
			3.73		+5.07	
			3.60		+1.41	
		1273	38.66	39.33	−1.70	0.74
			39.73		+1.02	
			39.60		+0.69	
47.51	52.49	1073	3.73	3.8.	−2.61	6 × 10^−2^
			3.73		−2.61	
			4.00		+4.44	
		1273	45.60	45.29	+0.68	–
			46.00		+1.57	
			44.00		−2.84	
60.00	40.00	973	0.80	0.97	−17.52	–
			0.80		−17.52	
			1.07		+10.31	
		1273	53.33	54.28	−1.75	–
			55.33		+1.93	
			54.13		−0.28	
					|Δ_av._| = 3.88	

**Table 4:** Vapor pressure of selenium and antimony selenide in the Sb_2_Se_3_–Se system.

Alloy Composition, at. %		*T*, K	${\bar{p}}_{Se}$, Experiment, kPa	${\bar{p}}_{Se}$, Calculation, kPa	Δ, rel. %	${\bar{p}}_{Sb_{2}Se_{3}}$, Calculation, kPa
Se	Sb					
68.96	31.04	873	14.00	14.30	−2.10	0.10
			14.40		+0.70	
			15.20		+6.29	
		973	57.33	57.17	+0.28	0.71
			58.39		+2.13	
			57.46		+0.50	
75.37	24.63	823	8.53	8.92	−4.37	3 × 10^−2^
			9.06		+1.57	
			9.20		+3.14	
		923	41.20	42.56	−3.20	0.24
			42.66		+0.23	
			42.13		−1.01	
83.38	16.62	773	3.87	4.20	−7.86	6 × 10^−2^
			4.00		−4.76	
			4.53		+7.86	
		873	23.86	25.12	−5.02	7 × 10^−2^
			24.66		−1.83	
			25.86		+2.95	
91.30	8.70	773	4.40	4.66	−5.58	4 × 10^−2^
			4.93		+5.79	
			4.67		+0.21	
		873	28.00	28.77	−2.68	5 × 10^−2^
			29.33		+1.95	
			29.46		+2.40	
100	–	773	4.93	5.33	−7.50	–
			5.60		+5.07	
			5.47		+2.63	
		873	33.33	34.00	−1.97	–
			34.40		+1.18	
			34.26		+0.76	
					|Δ_av._| = 3.12	

**Table 5:** Vapor pressure of components in the Sb–Sb_2_S_3_ system.

Alloy Composition, at. %		*T*, K	${\bar{p}}_{Sb_{2}S_{3}}$, Experiment, kPa	${\bar{p}}_{Sb_{2}S_{3}}$, Calculation, kPa	Δ, rel. %	${\bar{p}}_{Sb}$, Calculation, kPa
S	Sb					
18.47	81.53	1123	3.07	3.35	−8.36	0.19
			3.47		+3.58	
			3.47		+3.58	
		1323	28.00	28.72	−2.51	1.71
			28.80		+0.28	
			29.33		+2.12	
34.11	65.89	1123	3.20	3.23	−0.93	0.20
			3.47		+7.43	
			2.93		−9.29	
		1323	33.06	33.69	−1.87	1.51
			33.60		−0.27	
			34.40		+2.11	
41.07	58.93	1123	3.20	3.23	−0.93	0.20
			3.20		−0.93	
			3.33		+3.10	
		1323	34.66	36.05	−3.86	1.34
			36.66		+1.69	
			36.80		+2.08	
49.33	50.67	1123	3.33	3.34	−0.30	0.17
			3.20		−4.18	
			3.47		+3.89	
		1323	39.60	39.83	−0.58	0.98
			38.66		−2.94	
			41.33		+3.77	
60.00	40.00	1073	1.47	1.68	−12.50	–
			1.73		+2.98	
			1.87		+11.31	
		1273	26.40	26.67	−0.74	–
			27.06		+1.46	
			26.53		−0.52	
					|Δ_av._| = 3.34	

**Table 6:** Sulfur vapor pressure in the Sb_2_S_3_–S system.

Alloy Composition, at. %		*T*, K	${\bar{p}}_{S}$, Experiment, kPa	$\ln{\bar{p}}_{S}=B-A\cdot T^{-1}$		Boiling Point (°C) at Pressure, kPa	
S	Sb						
				*B*	*A*	101.325	0.900
67.43	32.57	523	1.60	23.701	8569	431	234
			1.33				
			1.60				
		673	58.66				
			57.33				
			58.00				
76.09	23.91	523	1.50	23.758	8590	429	234
			1.50				
			1.60				
		673	59.33				
			59.59				
			59.86				
83.33	16.67	523	1.33	24.093	8818	429	237
			1.50				
			1.33				
		673	59.06				
			59.59				
			59.33				
94.12	5.88	523	1.50	23.940	8718	429	236
			1.50				
			1.33				
		673	58.93				
			59.33				
			59.19				
100	–	523	1.33	24.081	8811	429	237
			1.50				
			1.33				
		673	59.19				
			59.65				
			58.66				

**Table 7:** Changes in the partial and integral vaporization entropies of liquid alloys in the Sb–Se system.

Alloy Composition, at. %		$\Delta {\bar{S}}_{Se}^{V}$, J/(mol × K)	$\Delta {\bar{S}}_{Sb_{2}Se_{3}}^{V}$, J/(mol × K)	$\Delta {\bar{S}}_{Sb}^{V}$, J/(mol × K)	$\Delta S_{Sb-Se}^{V}$, J/(mol × K)
Se	Sb				
0	100	–	–	73.02 ± 4.00	73.02 ± 4.00
10	90	–	81.32 ± 4.46	71.28 ± 3.91	72.95 ± 4.00
20	80	–	90.42 ± 4.95	68.28 ± 3.74	75.66 ± 4.15
30	70	–	96.90 ± 5.31	63.67 ± 3.49	80.28 ± 4.40
40	60	–	101.32 ± 5.55	57.50 ± 3.15	86.71 ± 4.75
50	50	–	104.03 ± 5.70	49.25 ± 2.70	94.90 ± 5.20
60	40	–	105.93 ± 5.12	–	105.93 ± 5.12
70	30	96.59 ± 4.56	113.02 ± 5.33	–	108.92 ± 5.14
80	20	101.85 ± 4.81	122.86 ± 5.80	–	112.36 ± 5.30
90	10	106.02 ± 5.00	137.71 ± 6.50	–	113.94 ± 5.38
100	0	110.06 ± 5.19	–	–	110.06 ± 5.19

**Table 8:** Changes in the partial and integral vaporization entropies of liquid alloys in the Sb–S system.

Alloy Composition, at. %		$\Delta {\bar{S}}_{S}^{V}$, J/(mol × K)	$\Delta {\bar{S}}_{Sb_{2}S_{3}}^{V}$, J/(mol × K)	$\Delta {\bar{S}}_{Sb}^{V}$, J/(mol × K)	$\Delta S_{Sb-S}^{V}$, J/(mol × K)
S	Sb				
0	100	–	–	72.95 ± 3.60	72.95 ± 3.60
10	90	–	81.33 ± 4.02	71.03 ± 3.51	72.75 ± 3.59
20	80	–	91.03 ± 4.50	67.85 ± 3.35	75.58 ± 3.73
30	70	–	97.96 ± 4.84	62.90 ± 3.11	80.43 ± 3.97
40	60	–	103.51 ± 5.11	55.02 ± 2.72	87.35 ± 4.32
50	50	–	108.18 ± 5.34	40.48 ± 2.00	96.89 ± 4.79
60	40	–	112.17 ± 5.54	–	112.17 ± 5.54
70	30	104.39 ± 5.15	–	–	104.39 ± 5.15
80	20	104.39 ± 5.15	–	–	104.39 ± 5.15
90	10	104.39 ± 5.15	–	–	104.39 ± 5.15
100	0	104.39 ± 5.15	–	–	104.39 ± 5.15

**Table 9:** Changes in the partial and integral enthalpies of the vaporization of the Sb–Se system’s components.

Alloy Composition, at. %		$\Delta {\bar{H}}_{Se}^{V}$, kJ/mol	$\Delta {\bar{H}}_{Sb_{2}Se_{3}}^{V}$, kJ/mol	$\Delta {\bar{H}}_{Sb}^{V}$, kJ/mol	$\Delta H_{Sb-Se}^{V}$, kJ/mol
Se	Sb				
0	100	–	–	139.31 ± 7.63	139.31 ± 7.63
10	90	–	121.52 ± 6.66	138.81 ± 7.61	135.93 ± 7.45
20	80	–	128.06 ± 7.62	136.59 ± 7.49	133.75 ± 7.33
30	70	–	134.11 ± 7.35	132.25 ± 7.23	133.18 ± 7.30
40	60	–	138.41 ± 7.58	126.26 ± 6.92	134.36 ± 7.36
50	50	–	140.63 ± 7.71	119.71 ± 6.56	137.15 ± 7.52
60	40	–	141.43 ± 6.68	–	141.42 ± 6.68
70	30	97.96 ± 4.62	141.36 ± 6.67	–	130.51 ± 6.16
80	20	99.56 ± 4.70	140.31 ± 6.62	–	119.93 ± 5.66
90	10	101.84 ± 4.81	136.22 ± 6.43	–	110.44 ± 5.21
100	0	104.00 ± 4.91	–	–	104.00 ± 4.91

**Table 10:** Changes in the partial and integral enthalpies of the vaporization of the Sb–S system’s components.

Alloy Composition, at. %		$\Delta {\bar{H}}_{S}^{V}$, kJ/mol	$\Delta {\bar{H}}_{Sb_{2}S_{3}}^{V}$, kJ/mol	$\Delta {\bar{H}}_{Sb}^{V}$, kJ/mol	$\Delta H_{Sb-S}^{V}$, kJ/mol
S	Sb				
0	100	–	–	139.31 ± 6.88	139.31 ± 6.88
10	90	–	124.08 ± 6.13	138.20 ± 6.83	135.85 ± 6.71
20	80	–	134.07 ± 6.62	134.87 ± 6.66	134.60 ± 6.65
30	70	–	142.11 ± 7.02	129.11 ± 6.34	135.61 ± 6.70
40	60	–	148.35 ± 7.33	120.17 ± 5.94	138.95 ± 6.86
50	50	–	153.29 ± 7.57	105.15 ± 5.19	145.27 ± 7.18
60	40	–	156.92 ± 7.75	–	156.92 ± 7.75
70	30	73.26 ± 3.62	–	–	73.26 ± 3.62
80	20	73.26 ± 3.62	–	–	73.26 ± 3.62
90	10	73.26 ± 3.62	–	–	73.26 ± 3.62
100	0	73.26 ± 3.62	–	–	73.26 ± 3.62

## Data Availability

Data are contained within the article.
